# Propolis, Aloe Vera, Green Tea, Cranberry, Calendula, Myrrha and Salvia Properties against Periodontal Microorganisms

**DOI:** 10.3390/microorganisms10112172

**Published:** 2022-10-31

**Authors:** Luciene Cristina Figueiredo, Nathalia Freitas Figueiredo, Daniele Ferreira da Cruz, Gustavo Titonele Baccelli, Gabriela Espinoza Sarachini, Manuela Rocha Bueno, Magda Feres, Bruno Bueno-Silva

**Affiliations:** Dental Research Division, Guarulhos University, Guarulhos 07023-070, Brazil

**Keywords:** oral microorganisms, biofilm, periodontitis, natural products, *P. gingivalis*

## Abstract

The oral cavity harbors hundreds of microorganisms that may be uncontrolled and provoke several diseases. In this sense, periodontitis is a complex multifactorial disease with an essential microbial component in its etiology. Periodontal treatment involves mechanical control of the supra- and subgingival biofilm, but not all patients respond predictably to treatment. In this way, the biofilm chemical control helps in the reduction of periodontal pathogens during treatment or in the delay of bacterial re-colonization after scaling and root planning. Several products have been studied as adjunctive therapy and have shown promising results. Therefore, the present article reviews the biological effects of propolis, aloe vera, green tea, cranberry, calendula, myrrha and salvia that may support their use in the control of subgingival biofilm in patients with periodontitis. All the natural products cited above showed exciting results against microorganisms related to oral diseases, mainly periodontitis. These substances also have anti-inflammatory and antioxidant activities. The natural agents propolis, aloe vera, green tea, cranberry, calendula, myrrha and salvia demonstrated potential to be used as oral hygiene products, based on their antimicrobial and anti-inflammatory actions.

## 1. Introduction

The oral cavity harbors more than 700 microorganisms [1,2]. The molecular analysis of microbiological cultures revealed the microbial composition of communities varies in relation to the natural place that they live [3]. For example, the oral biofilm varies according to its environment. The ecological conditions of supra- and subgingival ambiances are markedly unique, promoting the organization of a highly diverse community [4]. At the beginning, commensal bacteria adhere to the tooth surfaces and are followed by other exogenous infective species [5]. However, if not removed by a constant regular oral hygiene, undisrupted biofilms are responsible for dysbiosis in the oral microbiota, promoting the transition from the biofilm that was once “healthy” to the now periodontopathogenic biofilm, with tissue destruction as a result of the exacerbated immunoinflammatory response. In vulnerable individuals, this first stage of disease called gingivitis may develop to periodontitis, which is a more intricate illness with a multifactorial pathogenesis. It is characterized by alveolar bone loss due to an inflammatory reaction of the tooth-supporting tissues [6].

The current therapeutic possibilities for periodontal diseases are focused on the control of the supragingival and subgingival oral microbiota, through the use of local and systemic antimicrobials [7,8], and also on the immunomodulation of the host response through agents with anti-inflammatory potential.

Currently, research is focused on the search for substitutes of natural origin for chemical antimicrobials. Thus, the aim of this manuscript is to review the biological, especially antimicrobial, effects of propolis, aloe vera, green tea, cranberry, calendula, myrrha and salvia that may support their use in the control of subgingival biofilm in patients with periodontitis.

## 2. Etiology, Pathogenesis and Treatment of Periodontitis

Periodontitis is an infectious and inflammatory disease associated with dysbiotic dental biofilm, which invades the subgingival environment and generates a persistent and unbalanced inflammatory response, which results in the destruction of tooth-supporting tissues [9,10]. It is an important public health problem as it is considered the sixth most prevalent chronic non-communicable disease, causes functional losses and has a plausible negative impact on general health [11,12]. Its main features include loss of periodontal tissue, clinically manifested through loss of clinical attachment and alveolar bone loss, presence of periodontal pockets and bleeding on probing [10].

The periodontopathogenic biofilm is a complex community of microorganisms composed of some bacterial species gathered in the yellow, green and purple complexes and the blue group that were associated with health. Meanwhile, the three species of the red complex, *Tannerela forsythia*, *Porphyronomas gingivalis* and *Treponema denticola*, were strongly associated with increased bleeding on probing and probing depth [13,14,15]. As there is a quantitative increase in bacterial species and a change in the proportion of these morphological types, important inflammatory changes are established in the periodontal tissue induced by the biofilm [13,16].

This advance in the understanding of the etiology of periodontal diseases was only possible due to the improvement of technologies for microbiological diagnosis. Methods such as immunoassays, polymerase chain reaction (PCR), deoxyribonucleic acid (DNA) probes and checkerboard DNA–DNA hybridization allowed the identification of species that were difficult to identify using traditional methods, such as bacterial culture [17,18]. This knowledge contributed to the emergence of a new model of pathogenesis, according to which periodontitis is initiated by a synergistic and dysbiotic microbial community [19]. Fundamental pathogens called “keystones” can increase the virulence of the entire microbial community, modulate the host response and consequently favor dysbiosis [19]. In addition, studies using bacterial DNA sequencing techniques have begun to identify new species that may be related to periodontal disease, as well as proving the association of known periodontal pathogens [20,21,22].

Components of the bacterial biofilm, such as lipopolysaccharides (LPSs) and toxins, initiate the immune–inflammatory response by activating host defense cells, which include polymorphonuclear cells (PMNs), thus triggering a response against microbial invasion. Activation of defense cells results in the production of inflammatory mediators such as cytokines, chemokines, prostaglandins and proteolytic enzymes that, in turn, alter connective tissue and bone metabolism [23]. The defense process against a bacterial infection is initially mediated by pro-inflammatory cytokines, such as tumor necrosis factor alpha (TNF) and interleukins (IL)-1, IL-6, IL-8 and IL-12, in addition to monocyte/lymphocyte infiltration [24,25]. In this sense, the dysbiosis of the subgingival biofilm, the primary etiological factor of periodontal disease, induces a chronic local inflammation causing up-regulation of osteoclastogenesis and consequent destruction of the bone tissue supporting the teeth. This shifts the bone remodeling process towards increased bone resorption. Osteoclastogenesis is activated by macrophage colony-stimulating factor, by the nuclear factor kappa B ligand (RANKL) released by osteoblasts and responsible for osteoclast activation, and also by inflammatory mediators, including cytokines and prostaglandins [26].

For the treatment of periodontitis to be considered successful, the chosen therapy must improve periodontal clinical parameters, in addition to promoting ecological change in the biofilm, making the microbial profile more compatible with periodontal health [17,27,28]. The therapy known as the gold standard for periodontitis treatment is scaling and root planning, as it can reduce the clinical parameters of depth on probing and bleeding on probing and favoring the gain of the clinical level of insertion and the change of the microbial profile [17].

Scaling and root planning consist of mechanical removal and disorganization of the supra- and subgingival biofilm, and its results have been observed since the first reassessment [27]. According to the authors, an important clinical improvement and the greatest reduction of pathogenic subgingival species occurred in the first 6 months after therapy, and the results remained stable until one year after treatment or improved modestly after that [27]. Haffajee et al. [29] showed a reduction in the mean counts of bacterial species in the orange and red complexes after periodontal treatment. The most significant decrease occurred after two weeks, but for some species, there was a continuous decrease at the three- and six-month assessments, with a slight increase at twelve months. In addition, these authors correlated a reduction in bacterial species levels with an improvement in probing depth and warned that an increase in these species could be associated with clinical worsening [29]. Therefore, a factor related to the success of the treatment is the establishment of periodontal maintenance therapy, which may be essential in consolidating the clinical and microbiological improvements achieved as a result of the initial therapy [27].

However, not all individuals with periodontitis are able to maintain the benefits achieved right after scaling and root planning (SRP) in the long term [7,27,30,31]. One of the main reasons is the presence of pathogens throughout the mouth, which can be found in the supragingival biofilm and in other oral niches, such as the tongue, oral mucosa and saliva [13]. Even in healthy sites, individuals with periodontal disease have higher proportions of pathogens when compared to those without the disease [13,17]. In this sense, several studies over the years have tested adjunctive therapies to SRP, such as systemic antibiotic therapy [8], host response immunomodulators [32] and also the chemical control of the supragingival biofilm as an alternative for local therapy. The mouthwashes, usually applied for chemical control of biofilm, can potentiate these clinical and microbiological benefits [17,33,34,35,36,37,38,39]. Thus, it is necessary to search for therapies that are capable of maintaining the results achieved for an extended period and reach all areas of the mouth that need an anti-infective treatment.

## 3. Chemical Control of Supragingival Biofilm

Mechanical biofilm removal is necessary for the prevention, treatment and post-therapy maintenance of periodontal diseases, either professionally or through manual control by the individual [40,41]. However, satisfactory cleanliness levels are not always achieved with manual brushing alone. A systematic review evaluated the effectiveness of biofilm removal in adults with gingivitis and concluded that mechanical plaque control was not wholly effective in reducing signs of inflammation [42]. Furthermore, tooth surfaces only represent a small percentage of the total mouth area [43]. Therefore, the use of antimicrobial agents can help control the supragingival biofilm because they are able to reach other oral niches and can delay the accumulation on the tooth surface [44,45].

The use of mouthwashes is one of the ways of chemical control of biofilms. These agents can act by promoting cell death, inhibiting bacterial reproduction or inhibiting cell metabolism [46,47]. Many chemical agents with antimicrobial properties are being studied as active principles to control the formation of dental biofilm. Examples of these agents include biguanides (chlorhexidine), quaternary ammonium compounds (cetylpyridinium chloride, benzalkonium chloride), detergents, essential oils, phenolic compounds (triclosan), enzymes (mutanase/glucanase, amyloglucosidase/glucose oxidase), metal ions (zinc, copper, tin) and plant extracts (sanguinarine) [33,48,49,50,51,52].

In this context, chlorhexidine is a highly alkaline cationic biguanide, making it practically insoluble. For this reason, it is used as a soluble salt, the most common being chlorhexidine digluconate [53]. It has been used in medicine since 1953. It is considered the gold standard among oral antiseptics, as it has several characteristics that favor its applicability in dentistry, such as a broad spectrum of action and high substantivity [42,50,53].

The chlorhexidine molecule has a positive charge that interacts with the negative charge of the bacterial cell wall, causing an osmotic imbalance of the microorganism. Depending on the dosage, it may exert a bactericidal or bacteriostatic effect [50]. When concentrations are greater than or equal to 0.12%, chlorhexidine causes cell death (bactericidal) due to precipitation and release of cytoplasmic content from both Gram-positive and Gram-negative bacteria, as well as aerobic and anaerobic bacteria. At lower doses, it causes alteration in cell wall integrity and releases low-molecular-weight products that prevent bacterial reproduction (bacteriostatic) [42,50,53].

When added to periodontal treatment, chlorhexidine results in a slightly more expressive reduction in probing depth [38] and less gingival bleeding [50]. A meta-analysis evaluated studies that associated the use of chlorhexidine with the treatment of individuals with chronic periodontitis [38]. Among the included studies, only three had the primary objective of evaluating the effect of chlorhexidine on periodontal parameters. These studies used mouthwash at 0.12% for a period between 40 and 60 days; they also showed clinical benefits with chlorhexidine [33,34,54]. In this sense, chemical control of the biofilm can be an effective alternative to potentiate mechanical therapy.

The problem with the continued use of chlorhexidine mouthrinses during periodontal treatment is the possibility of developing adverse effects. The most reported in the literature are extrinsic pigmentation of teeth, tongue, mucosa and restorations; taste alteration; burning sensation; supragingival calculus formation; and less frequent cases of allergy. However, these effects are dose-dependent and tend to decrease when concentration is reduced or disappear when the mouthwash is discontinued [50,55,56,57]. For this reason, formulations with lower doses or other active ingredients have been described in the literature in an attempt to show similar benefits but with a lower frequency of adverse effects associated with the use of chlorhexidine.

One of the most studied options as an active principle is cetylpyridine chloride. This cationic ammonium quaternary compound was first described in 1945, demonstrating bactericidal and bacteriostatic effects on oral bacteria. Over the years, several studies have shown its antimicrobial activity. Recently, cetylpyridine chloride was tested through an in vitro subgingival multispecies biofilm model related to periodontitis and proved to be as effective as chlorhexidine [52]. Due to this demonstration of antimicrobial activity, cetylpyridine chloride has been studied in several clinical situations, including as an adjunct to the treatment of gingivitis [37,58], periodontitis [39,59] and peri-implantitis [60].

Another current option is the search for new natural active principles for dental application (dentifrices and mouthwashes). There is an intense effort in the literature to find novel antimicrobials with the capacity to disrupt the subgingival multispecies biofilm. One of the primary sources of new compounds is natural products [61,62,63,64,65].

The related information in this review was compiled using scientific databases such as Elsevier, PubMed and Wiley Online. The keywords “propolis”, “aloe vera”, “green tea”, “cranberry”, “calendula”, “myrrha”, “salvia”, “antimicrobial” and “anti-inflammatory” were applied to extract the literature. The initial search period was the last 20 years, with the most recent publications prioritized whenever possible.

## 4. Natural Products

Since the introduction of penicillin, there has been a considerable increase in the number of classes of antibiotics available for the care of the world’s population. However, unfortunately, the occurrence of several adverse effects [66] justifies the scientific search for other antimicrobial agents. Among all new drugs approved by the Food and Drug Administration (FDA) or other equivalent entities in other countries, most of all new drugs (~75%) currently approved are from natural sources or derived from natural sources [67].

Among natural products, propolis has stood out since it presents various biological properties and its application in the cosmetics and food industries, where it is used as an ingredient in the formulation of several products [68,69,70,71]. Propolis is a resinous substance collected by bees from different parts of the plant, such as buds, flower buds and resinous exudates. It has a varied color and consistency and is used to close small cracks, embalm dead insects and protect the hive against the invasion of microorganisms [72]. The main constituents are phenolic compounds, represented by flavonoids, phenolic acids and their esters, which play an important role in the body, as they can exert antioxidant, anti-inflammatory, antimicrobial and other biological activities [73,74,75,76,77,78]. Several other activities of propolis have been described, such as hepatoprotective properties [79]; analgesic [80], antiparasitic [81] and estrogenic activities [82]; and the regeneration of cartilage and bones by stimulating the proliferation of chondrocytes [83]. Due to its chemical complexity, propolis is considered one of the most heterogeneous mixtures in natural sources.

Among the propolis-producing countries, Brazil stands out because it is a country with varied vegetation, and thus, several types of propolis can be found in its large extension. Due to geographic extension and plant chemical biodiversity, Brazilian propolis was initially classified into 12 groups based on its complex chemical profile, determined by the appearance and color of the extracts, UV–visible absorption spectrum and chromatography [84]. This wide variety of types of propolis is uncommon in the world and does not occur in other countries in America, Asia and Europe.

Brazilian propolis from the South and Southeast region demonstrated better antimicrobial activity and thus received more attention from scientists. Currently, other types of propolis, such as the geopropolis and organic propolis, initially not included in the first classification mentioned above, also have great prominence in the literature [74,85].

The propolis produced in the South region is known as green propolis, and it was the first to be recognized due to its potential application as an antimicrobial agent. Recently, Brazilian green propolis was found to impair gut microbiota dysbiosis by enhancing the Bacteroidetes/Firmicutes proportion in an animal study. Generally, the Firmicutes phylum is recognized as disease-associated, and thus its diminution is of great importance [86]. In addition, the chemical composition analysis of green propolis revealed the presence of several distinct bioactive compounds such as baccharin, p-coumaric acid, apigenin and trans-trans-farnesol.

According to a recent in vivo study, baccharin and p-coumaric acid decrease neutrophil migration and nitric oxide release. In addition, both compounds regulate cytokine production: baccharin directly reduces inflammatory cytokines, while p-coumaric acid increases the production of the anti-inflammatory IL-10 [87]. Moreover, baccharin provokes *P. gingivalis* membrane depolarization, increasing its permeability. Recently, this effect was suggested as a possible antimicrobial mechanism of action of green propolis on *P. gingivalis* [88].

Apigenin is found in many foods and plants, including green propolis. It has several pharmacological properties such as anti-inflammatory [89], antimicrobial and anticaries properties [90]. This compound has been shown to inhibit the development of *Candida albicans* [91] and *S. mutans*, in addition to being a potent inhibitor of glucosyltransferase enzymes, which produce glucans, a critical virulence factor of *S. mutans* in caries disease [90]. However, no reports were found in the literature regarding its possible inhibitory activity on periodontal disease and its pathogens.

Trans-trans-farnesol is a sesquiterpene alcohol compound commonly found in citrus fruits and propolis [90] with antimicrobial and anticaries properties. Its likely mechanism of antimicrobial action includes altering the membrane function and physiology, which was determined using an in vitro multispecies biofilm model [92].

In 2008, the propolis, popularly known as red propolis, found on the coast of Maceió, Alagoas, northeast of Brazil, had its botanical origin determined as *Dalbergia ecastophyllum*, a plant belonging to the Leguminosae family, the same family as soybeans and beans. Several of its isolated compounds, such as neovestitol and vestitol, are commonly found in plants of the legume family, which belongs to the botanical origin of red propolis [72]. Thus, this propolis has recently been highlighted due to its biological properties and isolated compounds neovestitol and vestitol.

The crude extract of Brazilian red propolis reduced the proportion of a group of microorganisms called orange complex [14], a group of pathogenic microorganisms associated with the health–disease transition and necessary for the red complex (pathogenic) to establish itself in the biofilm [93]. The red propolis also effectively reduced the red complex in mature already-formed multispecies biofilm [94].

Concerning red propolis bioactive compounds, neovestitol was first isolated in 1976 from an African leguminous plant [95]. It remained unnoticed until a few years ago when chemical studies revealed its presence in Cuban red propolis [96,97]. Vestitol is an isoflavone present in plants of the legume family. Firstly, this compound was identified in Cuban red propolis samples [96,97]. The natural combination of neovestitol–vestitol (60/30% respectively) inhibited the formation and development of *S. mutans* biofilm in vitro and reduced the development of dental caries in an in vivo study at a concentration of 800 µg/mL [98], and it showed a minimum inhibitory concentration of 50 µg/mL against *P. gingivalis* and *A. actinomycetemcomitans* [99], demonstrating its potential to inhibit the growth of essential microorganisms in the development of biofilm-associated periodontitis.

The use of propolis in dentistry has been associated with mouthwash due to its antibacterial and anti-inflammatory benefits [100]. Recently, a propolis-based mouthwash reduced the morning bad breath of individuals enrolled in a clinical trial [69]. In 2020, a systematic review evaluated clinical trials regarding the efficacy of propolis mouthwash and chlorhexidine. They concluded that propolis has excellent potential for reducing plaque and gingival inflammation. However, further studies are suggested on using propolis in mouthwashes due to bias (short follow-up time, poorly designed studies, no placebo group) [101].

Another natural agent is aloe vera or *Aloe barbadensis*, which is a cactus-like succulent plant that belongs to the Liliaceae family [102]. Cosmetic and medicinal products are made from the mucilaginous tissue in the center of the aloe vera leaf. Aloe vera is known to have several therapeutic benefits and has long been used as a remedy for various conditions such as sunburn, wounds, skin problems and digestive tract disorders [102,103,104]. The pharmacological benefits of aloe vera are attributed to its wound-healing effects; immunomodulatory activity; and anti-inflammatory, antioxidant and antimicrobial properties [103,104]. Recent studies showed that the major active compounds with potential anti-inflammatory researched in the last years are aloe-emodin, aloin, aloesin, amodin, and acemannan [105](58). The potential of aloin to inhibit cytokines, ROS production and the JAK1-STAT1/3 signaling pathway is well documented [106,107].

Moreover, Li et al. [108] reported that aloe-emodin sulfates/glucuronides (0.5 μM), rhein sulfates/glucuronides (1.0 μM), aloe-emodin (0.1 μM),and rhein (0.3 μM) can inhibit pro-inflammatory cytokines, nitric oxide production, iNOS expression and MAPK phosphorylation. In another study, Thunyakitpisal et al. [109] demonstrated that acemannan increased IL-6 and IL-8 expression and NF-κB/DNA binding in human gingival fibroblast via a toll-like receptor signaling pathway. Na et al. [110] revealed that human saliva samples with high IL-1β content stimulated IL-8 production in KB cells, and pretreatments with aloin inhibited IL-8 production by decreasing the p38 and extracellular signal-regulated kinase pathway. With the advancement and growing popularity of herbal medicine in dentistry, aloe vera has been introduced in recent years for the treatment of various dental and oral conditions, including oral lichen planus, oral submucosal fibrosis, aphthous stomatitis, periodontitis and gingivitis, without the presence of side effects [102,104,111,112]. Several clinical trials have evaluated the effect of aloe vera mouthwash on biofilm and gingivitis, and the results of many studies support the use of aloe vera mouthwash as an effective substitute for chlorhexidine [113,114,115,116,117]. Aloe vera is also an option in toothpaste considering the antimicrobial potential on oral microorganisms, such as *Streptococcus mutans* and *Candida albicans*, and improvement in plaque index comparable to those obtained with products with triclosan in the composition [118,119,120].

Several components of green tea can also promote health benefits. Consumption of green tea beverages with a high bioactive compound content regulates the inflammatory processes by suppressing gene and protein expression of inflammatory cytokines [121]. Green tea contains four main catechins, epicatechin (EC), epicatechin-3-gallate (ECG), epigallocatechin (EGC) and epigallocatechin-3-gallate (EGCG); the latter is the most active and abundant [121,122]. Caffeine is a powerful antioxidant compound that is responsible for the antioxidant potential of the beverage [123]. Phenolic acids are secondary plant metabolites characterized by high antioxidant and anti-inflammatory potential, in addition to neuroprotective and hypoglycemic effects [124]. Other compounds found in green tea are rutin, quercetin and chlorophyll. Rutin, a polyphenolic compound, is a potent antioxidant and anti-inflammatory agent [125], quercetin is a phytochemical with antioxidant and neuroprotective activity [126], and chlorophyll and its derivatives exhibit strong antioxidant and anti-inflammatory activity [127]. The general anti-inflammatory properties of green tea include the ability to decrease protein denaturation and increase the production of anti-inflammatory cytokines. Its antioxidant properties can limit the number of free radicals, up-regulating basal levels and increasing the activity of these antioxidant enzymes [128].

Green tea has been shown to combat microorganisms in various ways, directly and indirectly, and has worked synergistically with some antibiotic agents. Its anti-inflammatory and antioxidant effects may also contribute to the antimicrobial effect [122]. Several studies have reported that green tea is effective against caries and periodontal disease, diseases with a strong correlation to microorganisms’ accumulation. [129]. In 2021, Mazur et al. [130] demonstrated through a systematic review that clinical periodontal parameters were found to be positively affected by green tea. Based on these results, there is sufficient evidence to support using green tea to prevent and treat periodontal disease.

More recently, in 2022, Kong et al. [131] published a literature review showing the antimicrobial activity of EGCG, a compound found in green tea, in the microbiota associated with oral diseases. Although the antimicrobial effect was evident for *P. gingivalis*, *A. actinomycetemcomitans*, *P. intermedia* and *F. nucletaum*, the authors highlight the need for new clinical studies to confirm the laboratory findings.

Cranberry is a fruit originally from England, grown throughout the Eastern and Northeastern United States and much of Canada. Research about cranberry health benefits began in the 1980s but has intensified and evolved rapidly over the past 25 years. In addition to the fruits, cranberry leaves can also be used to treat urinary disorders, diarrhea and diabetes. Cranberry contains several bioactive compounds, mainly polyphenols, including proanthocyanidins, anthocyanins, flavonols and phenolic acids. Cranberries are among some foods that contain type A proanthocyanidins, which have higher bioactivity compared to type B. Cranberry bioactive agents have unique characteristics, and modern science is trying to identify the connections between the health benefits and the specific compounds that this fruit has [132]. Particularly in dentistry, proanthocyanidins are known to inhibit oral biofilm adherence and for their anti-inflammatory effect that could potentially be able to neutralize the destructive inflammatory response of macrophages [133].

In the last year, several studies evaluated the anti-inflammatory/immunomodulatory potential of cranberries in periodontal or peri-implant therapy [134,135,136,137], demonstrating that their anti-inflammatory effect could potentially neutralize the destructive inflammatory response of macrophages [133]. As the principal anti-inflammatory effect, the proanthocyanidin compounds down-regulated the NF-κB signaling pathway. They decreased IL-6, IL-8, TNF-α and PGE_2_ production by distinct cell types such as gingival epithelial cells, neutrophils and macrophages [135,138]. In addition, a recent review detailed the antioxidant activity and the impact on human health based on the use of cranberry. Compounds from cranberry scavenge free radicals, the sole electrons in their external orbit, and then remove the reactive oxygen species (ROS) that react with biological membranes. Oxidative stress is caused by an excess of ROS in the biological fluids of the human body which is related to several illnesses. Therefore, compounds with antioxidant properties may be able to prevent or diminish oxidative damage to bacterial cell wall composition [139]. Therefore cranberries represent a rich source of phenolic acids and flavonoids linked to various health benefits [139].

*Calendula officinalis*, a herbaceous plant of the Tagetes genus and Asteraceae family, is native to Mexico and commercially cultivated as a popular ornamental plant for its broad spectrum of attractively colored, shaped and sized flowers [140,141]. In recent years, its medicinal potential has encouraged scientific studies in dentistry, especially on topics involved in the treatment of periodontitis and peri-implantitis [142,143,144].

*C. officinalis* possesses many secondary metabolites (triterpenoids, flavonoids, coumarins, quinones, volatile oil, carotenoids and amino acids) with various pharmacological properties [145,146,147]. Givol et al. [147] highlighted the anti-inflammatory action of the triterpenoids and flavonoids. The data suggest that *C. officinalis* reduces inflammatory bone resorption in experimental periodontitis, which may be mediated by its anti-inflammatory properties and its effects on bone metabolism, by decreasing neutrophilia and the expression levels of pro-inflammatory mediators TNF-α, IL-1β and RANKL. In addition, it increased the number of cells immunopositive for osteoprotegerin (OPG), an antiresorptive molecule.

The literature about calendula antimicrobial activity is poor. Only one report was found, which demonstrated that a calendula-based dentifrice did not present an antimicrobial effect on *A. viscosus*; *C. albicans*; *L. casei*; *S. mitis*; *S. mutans*; S. *oralis*; *S. sanguis*; *S. sobrinus*; and clinically isolated *C. albicans*, *S. mitis*, *S. mutans*, *S. oralis*, *S. sanguis*, *S. sobrinus* and *Lactobacillus* sp. [148].

The medicinal plant *Commiphora myrrha* (family Burseraceae) produces the aromatic oleo-gum-resin, known as myrrha. The oleo-gum-resin of *C. myrrha* is one of the most known natural antimicrobial agents, mainly due to its furanosesquiterpenes [134]. The genus Commiphora includes over 150 species of trees and shrubs primarily located in Africa, India, Yemen and the southern regions of Saudi Arabia. This natural product has been used to treat a variety of diseases, such as amenorrhea, ache, dysmenorrhea, fever, stomach complaints and gallbladder disease [149]. The gum of myrrha consists of polysaccharides and proteins, whereas the volatile oil contains steroids, sterols and terpenes. The crude extracts exhibit diverse biological activities, such as anesthetic and anti-inflammatory activities [150].

Regarding antimicrobial action, Ebani et al. [151] evaluated the in vitro antimicrobial activity of some essential oils because of a potential cutaneous application. *C. myrrha* essential oil was effective against five tested isolates (*Staphylococcus aureus*, *Staphylococcus pseudointermedius*, *Staphylococcus hyicus* and two *Staphylococcus chromogenes*) with 10 mg/mL MIC, and it was not active against the three tested *S. xylosus* strains [151].

In 2016, similar results were found by Mahboubi and Kashani [152] that related the relevant activity of *C. myrrha* against a *S. aureus* ATCC strain. Al-Marby et al. [153] evaluated the antimicrobial activity of methanol extracts from 17 plants used in ethnopharmacology and ethnomedicine around the tropics and sub-tropics, particularly in Saudi Arabia and Yemen. Among them, the properties of *C. myrrha* were also investigated. *Steinernema feltiae*, *Staphylococcus carnosus*, *Escherichia coli* and *Saccharomyces cerevisiae* were used as test organisms. The results demonstrated that extracts of *C. myrrha* and *C. murale* had the most active antibacterial activity with inhibition zones of 12 and 15 mm and minimum inhibitory concentrations of 2.5 mg/mL for both bacteria; however, the antifungal activity showed no relevance [153].

The action of myrrha in the prevention of diseases and maintenance of oral health has been studied for some time [154]. In the same direction, silver nanoparticles (SNs) have been studied for their antibacterial effect in different buccal uses, including mouthwash, being added to dental resin, and toothpaste [155,156,157]. In this context, recently, ALHarthi et al. [158] compared the antimicrobial activity of myrrha mixed with SNs against *P. gingivalis* and these solutions in their separate forms. Data analysis showed that myrrha mixed with SNs displayed superior antimicrobial activity against *P. gingivalis* compared to myrrha solution and SNs after 48 h of incubation [158].

The genus Salvia is another natural product with several biological properties related in the literature, mainly antimicrobial. Among the various salvia species, one of the most studied regarding its therapeutic potential is *Salvia officinalis*. It is a member of the family Lamiaceae and a traditional medicinal herb characterized as a low perennial shrub originating in the Mediterranean region. Its family is known to comprise over 900 species [159]. The *S. officinalis* essential oil contains major terpenes such as manool, eucalyptol, borneol and thujone. Other substances have also been identified in satisfactory concentrations: carnosol, carnosic acid, rosmarinic acid, flavonoids, polysaccharides, tannic acid, oleic acid, ursonic acid, ursolic acid, fumaric acid, chlorogenic acid and caffeic acid [160,161]. The data obtained from studies indicate that *S. officinalis* is an important antioxidant and anti-inflammatory agent [162,163]. Several researchers have tested the antimicrobial activity of *S. officinalis* against various microorganisms: *S. mutans* [164,165,166,167]; *S. aureus*, *S. epidermidis* and *C. albicans* [164]; *Lactobacillus casei* [165,166,167]; and *P. gingivalis* [168].

Mendes et al. [168] analyzed the microbiological activity of the *S. officinalis* crude extract, partitions, fractions and pure substances by means of five antimicrobial tests against six clinical isolates and three standard strains of periodontopathogens: *P. gingivalis*, *A. actinomycetemcomitans*, *P. intermedia*, *Prevotella nigrescens*, *F. nucleatum* and *Prevotella melaninogenica*. The results showed that S. officinalis crude extract has moderate action against all the periodontal bacteria tested in this study, with *A. actinomycetemcomitans* being the most sensitive and *P. gingivalis* being resistant to all the tested samples [168]. Marutescu et al. [169] also demonstrated the antimicrobial activity of essential oils from *S. officinalis* against Gram-positive and Gram-negative bacterial strains isolated from the oral cavity of patients with periodontitis using an in vitro analysis.

Based on the above, a novel gel (Desplac Gel Oral Premium) for oral use was developed and includes a range of natural products (propolis, aloe vera, green tea, cranberry, calendula) in its chemical composition. As an example of the use of novel microbiology technologies, our research group tested its antimicrobial activity through a multispecies biofilm model related to periodontitis. In this manner, studies employing oral biofilm models are particularly valuable [170,171,172]. The in vitro biofilm models, particularly the multispecies ones, and molecular assays of clinical trials provided the current knowledge about the microbiology of periodontitis [172]. The biofilm was exposed to Desplac and a placebo from the beginning of its formation, as described below.

## 5. Antimicrobial Property Measurement in a Multispecies Biofilm Model

The following bacterial species were used: Actinomyces gerencseriae ATCC 23840, Actinomyces israelii ATCC 12102, Actinomyces naeslundii ATCC 12104, Actinomyces oris ATCC 43146, Actinomyces odontolyticus ATCC 17929, Veillonella parvula ATCC 10790, Streptococcus gordonii ATCC 10558, Streptococcus intermedius ATCC 27335, Streptococcus mitis ATCC 49456, Streptococcus oralis ATCC 35037, Streptococcus sanguinis ATCC 10556, Streptococcus anginosus ATCC 33397, Streptococcus mutans ATCC 25175, Aggregatibacter actinomycetemcomitans ATCC 29523, Capnocytophaga gingivalis ATCC 33624, Capnocytophaga ochracea ATCC 33596, Capnocytophaga sputigena ATCC 33612, Eikenella corrodens ATCC 23834, Campylobacter gracilis ATCC 33236, Campylobacter rectus ATCC 33238, Campylobacter showae ATCC 51146, Eubacterium nodatum ATCC 33099, Eubacterium saburreum ATCC 33271, Fusobacterium nucleatum subsp. polymorphum ATCC 10953, Fusobacterium nucleatum subsp. vincentii ATCC 49256, Fusobacterium periodonticum ATCC 33693, Parvimonas micra ATCC 33270, Prevotella intermedia ATCC 25611, Streptococcus constellatus ATCC 27823, Tannerella forsythia ATCC 43037, Porphyromonas gingivalis ATCC 33277, Gemella morbillorum ATCC 27824, Propionibacterium acnes ATCC 11827 and Selenomonas noxia ATCC 43541.

The multispecies biofilm model was developed using the Calgary Biofilm Device, which is composed of a 96-well plate (Nunc system; Thermo Scientific, Roskilde, Denmark) and a lid containing the polystyrene pegs. The bacteria species were allowed to adhere to the polystyrene pegs to form the multispecies biofilm on them. At the beginning of the first day of biofilm formation, the placebo and the natural product Desplac were placed into the wells to evaluate their effects on biofilm formation. After seven days of biofilm formation, the pegs were collected, and the metabolic activity and the checkerboard DNA–DNA hybridization were performed [93,173,174].

**Figure 1:**
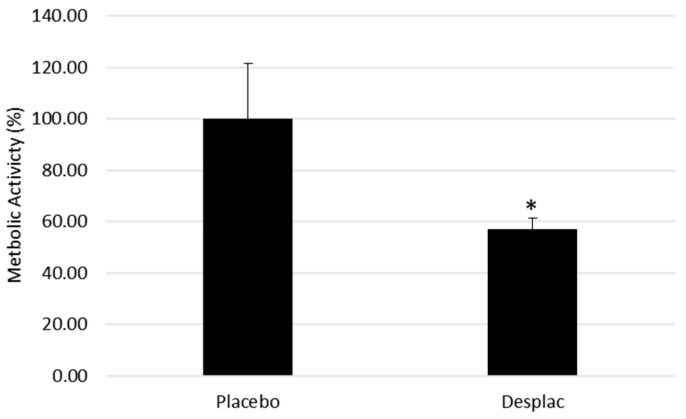
Mean and standard deviation of the mean of the metabolic activity of biofilms treated with the different agents. * A statistically significant difference using ANOVA, followed by Tukey’s test (*p* ≤ 0.05).

The percentage of metabolic activity reduction of the biofilms was determined using 2,3,5-triphenyl tetrazolium chloride (TTC) (catalog No. 17779; Fluka analytical, manufacturer, Buchs city, Switzerland) and spectrophotometry. TTC is utilized for the distinction between metabolically active and inactive bacterial cells. The white substratum is enzymatically reduced to a red formazan 1,3,5-triphenyl tetrazolium by bacterial live cells, due to the activity of several dehydrogenase enzymes, and then, the change in substrate color is read by spectrophotometry to determine the rate of reduction. The reduction in biofilm metabolic activity by Desplac was approximately 43% greater than that of the placebo group (Figure 1)

**Figure 2:**
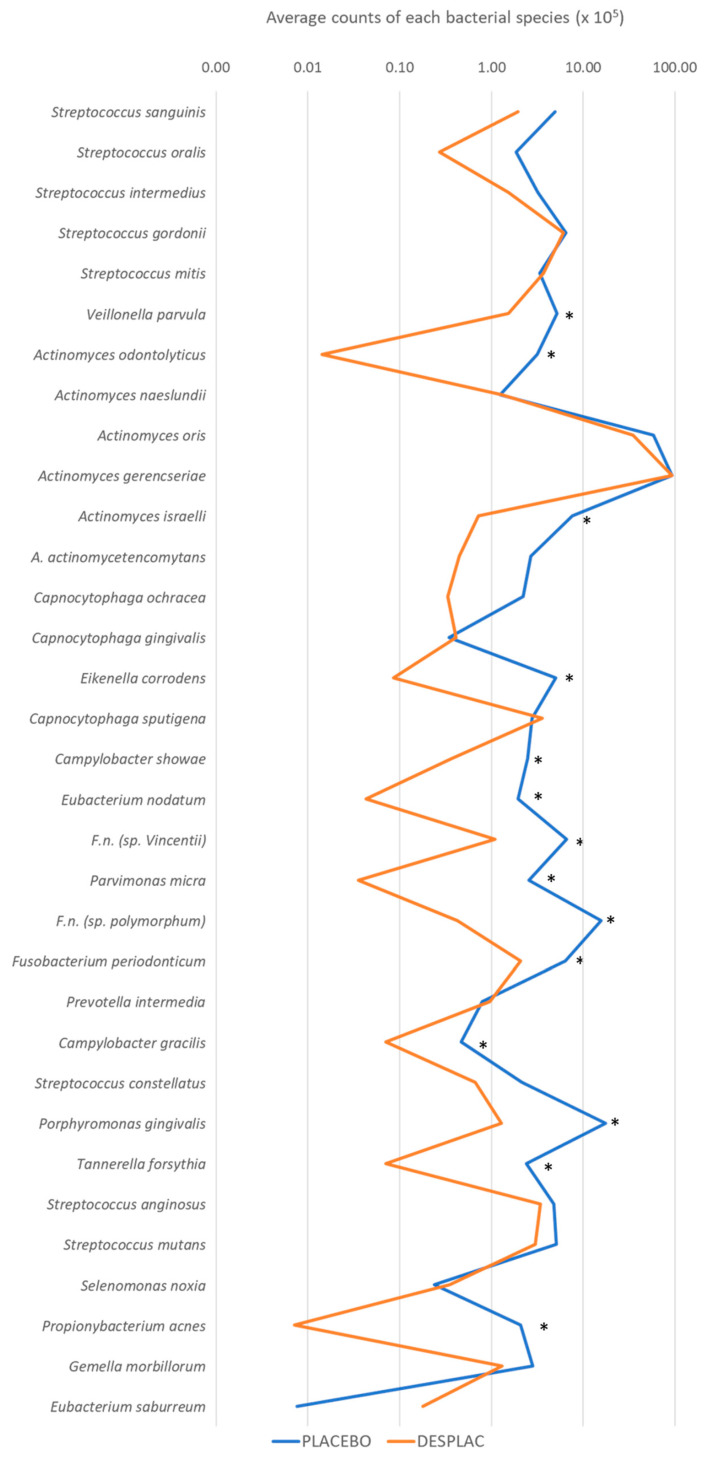
Average counts of each of the bacterial species present in the biofilm model under placebo and Desplac treatments. Statistical analysis performed using the Kruskal–Wallis test followed by Dunn’s post hoc test (*p* ≤ 0.05). * Statistical difference between placebo and Desplac.

The biofilm samples treated with the product and the placebo were analyzed individually for the presence and quantity of all bacterial species included in the model, using the DNA–DNA hybridization technique. In brief, the samples were lysed, and the DNA was positioned into a nylon membrane employing a Minislot device (Immunetics, Cambridge, MA, USA). Digoxigenin-labeled DNA probes from the complete genome of the subgingival bacterial species used were hybridized to single tracks of Miniblotter 45. After hybridization, the membranes were rinsed, and the DNA probes were identified employing an antibody specific to digoxigenin conjugated with phosphatase alkaline. The signals were acquired utilizing an AttoPhos substrate (Amersham Life Sciences, Arlington Heights, IL, USA), and the results were achieved using a Typhoon Trio Plus (Molecular Dynamics, Sunnyvale, CA, USA). Two lanes in each apparatus comprised the standards with 105 and 106 cells of each bacterial species. Signals acquired with the Typhoon Trio were converted to absolute counts by comparison with the standards on the same membrane. Failure to detect a signal was recorded as zero. The values obtained for each bacterial species in biofilm treated with the product were compared to those of biofilms treated with the placebo. Figure 2 shows the individual mean count of each bacterial species included in biofilm formation as assessed by checkerboard DNA–DNA hybridization. Desplac reduced the count of 14 different bacteria compared to the placebo-treated group, highlighting three species of *Fusobacterium*, *P. micra*, *P. gingivalis* and *T. forsythia*.

*P. gingivalis* and *T. forsythia* are species that orchestrate microbial dysbiosis and contribute to the mechanisms by which microorganisms avoid the human immune system. Both are members of the disease-associated red complex [5,14]. The species of Fusobacterium and *P. micra* are members of the orange complex and very common targets in periodontal research [175,176,177]. The inhibition of members of the orange complex is a very exciting result, which was not obtained even upon chlorhexidine treatment in previous articles from our research group [93].

The orange complex is correlated with the health–disease shift since the species in this complex generate beneficial conditions for the red-complex species to establish and succeed in the subgingival microbiome. Therefore, if the amounts of orange-complex members decrease on subgingival sites, they do not benefit the red-complex bacterial species virulence factors and establishment [14,15]. The diminution in the levels of these species indicates that a product with natural products as an active principle may be helpful to avoid bacterial re-colonization after periodontal treatment.

## 6. Final Remarks

According to the World Health Organization, most of the world’s population uses medicinal plants to relieve the symptoms of various clinical conditions. Based on ethnopharmacological studies, medicinal plants began to have scientific support. Traditional medicine has been rescued, with the aid of the World Health Organization, as it has always been a culturally widespread therapeutic alternative in the pursuit of health promotion. Natural products can be taken orally, in the form of powder for dilution, gels, infusions or teas. Topically, they are presented in the form of water- or oil-based preparations. One of the forms of the therapeutic use of plants is through their essential oils, also called volatile oils. These can be obtained from different plant materials such as flowers, leaves, fruits and roots. Originating from the secondary metabolism of these plants, they can be an excellent form of topical application due to their reasonable absorption rate. In addition, the adverse and side effects of natural products are less aggressive, providing better health conditions for the population. This affirmation does not mean that phytotherapeutics do not cause risks to the organism, and there is a need for research on their quality, efficacy and safety of use.

Natural products are a more sustainable and ecological alternative. This is because they are biodegradable and free of components that are harmful to the environment. Some of the main benefits arising from the use of natural products include the following: formulas that are not aggressive to the human body; plant components; medicinal properties; absence of toxicity; not presenting polluting agents in nature; decreasing the risk of allergies and inflammatory diseases.

The population looking at consumption of foods of natural origins has been increasing every year, and because of that, the development of new commercial products by the industry is also increasing. Although the literature demonstrates biological plausibility for using various natural agents, it is necessary to carry out robust scientific clinical studies that prove and/or support the commercial indications of these products.

The critical challenge in discovering novel natural agents is separating and purifying enough quantities of active principles (with high purity) from chemically complex crude extracts. The biological outcome must be associated with the bioactive molecule, and adequate methods for the separation of compounds should be developed to yield the required amount of the active agent.

Considering the oral ecosystem, periodontal disease is a worldwide disease whose main etiological factor is the dysbiosis of subgingival biofilm associated with an exacerbated inflammatory response. The literature demonstrated the potential of several natural products for dental applications. Propolis, aloe vera, green tea, cranberry, calendula, myhrra and salvia stand out and have tremendous potential to be used as antimicrobials within oral hygiene products. However, the absence of well-designed clinical trials appears as the main limitation to the use of these natural products. Therefore, future studies regarding the antimicrobial and anti-inflammatory properties of these products should focus on their clinical use. Finally, these natural products should be considered for commercial oral hygiene product development in the future.

## 7. Conclusions

The natural agents propolis, aloe vera, green tea, cranberry, calendula, myrrha and salvia demonstrated potential to be used as oral hygiene products based on their antimicrobial and anti-inflammatory actions.

## Data Availability

Not applicable.
